# Machine Learning and Multidrug-Resistant Gram-Negative Bacteria: An Interesting Combination for Current and Future Research

**DOI:** 10.3390/antibiotics9020054

**Published:** 2020-01-31

**Authors:** Daniele Roberto Giacobbe, Sara Mora, Mauro Giacomini, Matteo Bassetti

**Affiliations:** 1 Department of Health Sciences (DISSAL), University of Genoa,16132 Genoa, Italy; matteo.bassetti@unige.it; 2 Clinica Malattie Infettive, Ospedale Policlinico San Martino—IRCCS, 16132 Genoa, Italy; 3 Department of Informatics Bioengineering, Robotics, and Systems Engineering (DIBRIS), University of Genoa, 16145 Genoa, Italy; sara.mora.1994@gmail.com (S.M.); Mauro.Giacomini@dibris.unige.it (M.G.)

**Keywords:** antimicrobial resistance, Gram-negative, MDR, machine learning

## Abstract

The dissemination of multidrug-resistant Gram-negative bacteria (MDR-GNB) is associated with increased morbidity and mortality in several countries. Machine learning (ML) is a branch of artificial intelligence that consists of conferring on computers the ability to learn from data. In this narrative review, we discuss three existing examples of the application of ML algorithms for assessing three different types of risk: (i) the risk of developing a MDR-GNB infection, (ii) the risk of MDR-GNB etiology in patients with an already clinically evident infection, and (iii) the risk of anticipating the emergence of MDR in GNB through the misuse of antibiotics. In the next few years, we expect to witness an increasingly large number of research studies perfecting the application of ML techniques in the field of MDR-GNB infections. Very importantly, this cannot be separated from the availability of a continuously refined and updated ethical framework allowing an appropriate use of the large datasets of medical data needed to build efficient ML-based support systems that could be shared through appropriate standard infrastructures.

## 1. Introduction

Multidrug-resistant Gram-negative bacteria (MDR-GNB) have now become endemic in several countries [1,2,3,4]. From a clinical perspective, the dissemination of MDR-GNB carries an increased risk of inadequately treating patients with severe infections caused by these organisms, in part for the following two reasons: (i) as per definition, MDR-GNB are concomitantly resistant to multiple classes of antibiotics, thereby increasing the chance of selecting an inactive empirical treatment, and (ii) even once susceptibility test results become available (usually 48–72 h after the onset of the disease and the initiation of empirical treatment), their targeted treatment could remain suboptimal, since the MDR phenotype may force clinicians to choose among very few remaining options, including nephrotoxic agents or agents with impaired pharmacokinetics in some sites of infection [5]. Fortunately, some novel antibiotics have recently become available, that have enriched our armamentarium and have improved our ability to effectively counteract some of these deadly infections [6,7,8,9]. Nonetheless, this renewed availability of effective agents raises another important need, that of preserving the activity of these novel agents in the long term, by using them wisely according to antibiotic stewardship principles [10].

Overall, this complex scenario has inspired three important lines of clinical research to ultimately help physicians dealing with MDR-GNB in clinical practice: (i) identification of patients at risk of MDR-GNB infections (endpoint: development of MDR-GNB infection), (ii) identification of the best interventions (treatments) to improve the prognosis of MDR-GNB infections (endpoints: clinical cure/mortality), and (iii) identification of interventions (infection-control and antimicrobials stewardship measures) to counteract emergence and dissemination of resistance (endpoints: prevalence of resistance/incidence rates of MDR-GNB infections).

To these aims, clinical research mostly relied (and relies) on classical predictive models, which have been able to provide very useful risk scores to assist clinicians’ choices at the bedside of patients at risk of (or with) MDR-GNB infections [11,12,13]. Nonetheless, in recent years there has also been an increasing interest in exploiting the aid of machine learning (ML) techniques to better assist clinicians in complex bedside tasks. Indeed, starting from patients’ data, ML clinical decision support systems are able to learn the association between patients’ characteristics (input features of ML algorithms) and the endpoints of interest (output labels of ML algorithms). Notably, this is not dissimilar from what classical statistical models do (i.e., pointing out an association between patients’ characteristics and a given outcome measure). Consequently, classical and ML-based risk prediction through ML may be viewed as partly overlapping techniques, with the distinction between their aims and application being not always so clear. Nonetheless, this overlapping is not necessarily a disadvantage, and in our opinion, these two approaches should be ultimately viewed as complementary (to exploit their respective advantages according to the characteristics of any given task and the magnitude of the available data).

In this narrative review, we address the potential role of ML algorithms for assessing three different types of risk: (i) the risk of developing a MDR-GNB infection, (ii) the risk of MDR-GNB etiology in patients with an already clinically evident infection, and (iii) the risk of anticipating the emergence of MDR in GNB through the misuse of antibiotics. We will employ a case study approach, i.e., by selecting and discussing one existing example of the implementation of ML algorithms for each type of the three risks mentioned above.

## 2. Methods

In October 2019 we performed a PubMed search, employing the following search strings: (i) machine learning AND Gram-negative, and (ii) machine learning AND (antimicrobial resistance OR antimicrobial stewardship). By means of title and abstract screening, from an initial total of 247 papers we first selected only those studies pertaining to the use of ML algorithms for possibly guiding clinical decisions. Then, following evaluation of full texts and references of selected papers, we ultimately retained only those studies investigating the use of machine learning for measuring/classifying the risk of either developing or having an infection due to MDR-GNB, or of prompting the emergence of resistance. Eventually, we collectively selected a single pertinent example for narratively discussing each of the three topics introduced above.

The present review is structured in the following sections: (i) ML in brief, (ii) ML algorithms for predicting the risk of developing MDR-GNB infections, (iii) ML algorithms for predicting the risk of MDR-GNB etiology in patients with sign and symptoms of infection, and (iv) ML algorithms to guide antibiotic stewardship and preventing further emergence of MDR-GNB.

## 3. ML in Brief

ML is a branch of artificial intelligence that consists of conferring on computers the ability to learn from data [14,15]. Differently from classical computer expert systems, which are explicitly programmed to do specific task/s (e.g., recognizing a patient to be at risk of MDR-GNB infection), ML algorithms have a notable advantage in term of flexibility, since they may be able to point out the association between patients’ characteristics (the input) and the risk of MDR-GNB infection (the output) without being explicitly programmed to do so [16].

As anticipated in the introduction, this overlaps with what classical statistical risk prediction models do, since they also are able to predict associations between specific data and specific outcomes by employing/adapting different models (e.g., linear regression, logistic regression, Cox regression, or other models, dependent of the type of data and the nature of the assessed endpoint/s). It is thus not surprising that most introductory courses on ML start with lessons on linear regression and logistic regression.

Consequently, classical statistical models and ML algorithms should be considered as a continuum in which, generally, the fewer the assumptions imposed by humans (e.g., human-made strict selection of variables to be included in the model) the more likely it is for ML algorithms to capture complex and composite characteristics (features) not easily recognized by humans, and to evaluate their association with the output label/s with high accuracy [17]. In this regard, two important concepts should necessarily be taken into account. First, capturing/designing complex features usually requires a far larger amount of data than usually needed for classical risk prediction (the fields of ML and “big data” are indeed deeply intertwined) [18]. Second, the general assumption made just above (i.e., less human guidance coupled with increased use of ML algorithms) should be viewed more as a background trend (over the continuum of classical statistical models and ML algorithms) rather than an absolute rule. Indeed, human involvement also remain crucial in some complex tasks performed by ML algorithms (as an important guidance for pre-processing data, defining the algorithm architecture, appropriately tuning the algorithm hyperparameters, reducing/eliminating biases, and preserving model interpretability). In addition, less complex ML algorithms and/or ML algorithms with more human guidance may be used in addition to classical statistical approaches for the analysis of more restricted databases, still possibly providing useful, complementary information.

In the following sections, we will further explore these concepts by reviewing some examples of existing studies that implemented ML algorithms in the field of MDR-GNB infections. In addition, different ML algorithms are schematically summarized in Table 1.

## 4. ML Algorithms for Predicting the Risk of Developing MDR-GNB Infections

In this section, we describe a case example of a recent study exploiting he use of ML algorithms for depicting the risk of colonization [19] by extended-spectrum β-lactamases (ESBL)-producing Enterobacterales (ESBL-PE), a type of MDR-GNB. Indeed, although colonization by MDR-GNB is not always followed by MDR-GNB infection (i.e., colonized patients may not develop infection), the latter usually follows the former. Consequently, identifying modifiable risk factors on which to intervene for reducing the risk of colonization is intuitively a good, indirect way to prevent MDR-GNB infection.

The study performed by Tacconelli and colleagues is a good example of combining classical statistical techniques and ML algorithms to provide useful complementary information [19]. First, the authors used flexible parametric survival models (adjusted for colonization pressure and ward of hospital stay) and identified previous antibiotic exposure as one of the independent predictors of ESBL-PE colonization (hazard ratio 2.38, 95% confidence intervals [CI] 1.29–4.40, *p* = 0.006). Then, they employed a random forest algorithm (a ML technique consisting of aggregating the results of separated decision trees, see Table 1) in order to translate a complex interaction of different features (number of used antibiotics, use of combinations of antibiotics, sequential use of antibiotics, length of antibiotic therapy, baseline patients’ characteristics) in a simple rank of different antibiotic classes in terms of their influence on the risk of developing ESBL-PE colonization. Eventually, this approach provided useful additional information, by ranking previous monotherapy to be more at risk of promoting ESBL-PE colonization than previous combination therapy, and cephalosporins to be the most influential single antibiotic class. Of note, the authors initially trained different ML algorithms (linear support vector machine (SVM) with radial basis function kernel, neural network, random forest, and others) and ultimately selected the random forest algorithm on the basis of the best performance on the cross-validation set in terms of accuracy, overfitting, and permutation significance [19]. This further testified to the role of thoughtful human guidance of optimizing the application of ML techniques to specific risk prediction tasks.

## 5. ML Algorithms for Predicting the Risk of MDR-GNB Etiology in Patients with Sign and Symptoms of Infection

At the bedside of a patient with sign and symptoms of infection, it is critical to recognize the risk of MDR-GNB etiology, since it allows prompt initiation of an empirical treatment also covering MDR organisms. This to avoid the increase in mortality associated with a delayed administration of an active therapy [20,21,22]. On the other hand, MDR-GNB-covering agents should not be used indiscriminately in patients not a risk, since their misuse may lead to the perpetuation of resistance selection [23,24].

In their multicenter, retrospective study among 1288 patients with Enterobacterales bacteremia, Goodman and colleagues employed a classical, multivariable logistic regression approach for creating a risk score based on regression coefficients for predicting the likelihood of ESBL-PE etiology at the bedside of bacteremic patients, and compared its performance with that of risk assessment using a classification and regression tree approach (CART analysis) [25]. Overall, the two approaches performed similarly in the study of Goodman and colleagues, with 49.5% sensitivity and 99.5% specificity for the classical risk score and 51.0% sensitivity and 99.1% specificity for the decision tree.

Regarding the use of both classical and ML models, in our opinion the most interesting point in this paper was not their possible complementarity, but, the difference in their respective advantages against a backdrop of similar overall performance, which may alternatively prioritize the use of one approach over the other in different situations. For example, from a research perspective, while the classical logistic regression approach requires more assumptions a priori and may struggle with collinearity, these are not limitations of CART analysis, which also usually allows higher predictor-to-event ratios than classical logistic regression models [25,26]. On the other hand, CART analyses could be more prone to overfitting than classical models (and consequently perform more poorly on new data) [27]. From a clinical perspective, the major advantage of the classical logistic regression-based approach is its adaptability (i.e., sensitivity or specificity may be preferably prioritized in any given situation by shifting the cut-off), while the major advantage of CART analysis is perhaps its user-friendliness (e.g., in Goodman’s study it was based on only five variables and did not require end user calculation, while the classical score required 14 variables to be considered, as well as the compilation of the final summary score by the user).

Overall, we think the study by Goodman and colleagues suggest that a careful balance of the peculiarities and disadvantages of the two approaches still remains critical both (i) to identify the most suitable model for a given research or clinical task, and (ii) to precisely evaluate if and how to exploit a combined approach to obtain precious complementary information.

## 6. ML Algorithms to Guide Antibiotic Stewardship and Prevent Further Emergence of MDR-GNB

Antibiotic stewardship may be defined as a coherent set of actions designed to use antimicrobials responsibly (for example, but not only, optimal selection, dose, and duration of therapy) in humans, animals, and the environment, within a One Health approach [28,29,30]. In hospitals, these actions are intended not only for improving treatment of the patient receiving (or not receiving) antibiotic/s, but also for preventing/delaying the emergence of resistance that may disseminate to other patients. From the perspective of this enlarged and complex target population, the impact of antibiotic stewardship interventions may be complex to evaluate.

Beaudoin and colleagues employed a two-step ML approach (temporal abstraction followed by temporal induction classification models) to build a clinical decision support system able to learn classification rules for labeling inappropriate antibiotic prescriptions (based on a training set of past stewardship recommendations on antibiotic dose adjustment, discontinuation, and de-escalation in a Canadian hospital) [31]. The performance of the learning module was then evaluated prospectively, alongside with that of the classical antibiotic stewardship intervention already implemented in the hospital. Interestingly, by expanding the rules for identifying inappropriate prescriptions, the learning module was able to label inappropriate prescribing practices (i.e., not supported by local experts) that were missed by the standard antibiotic stewardship intervention rules [31].

Although several limitations were identified by the authors (e.g., how to address alterations, substitutions, and maintenance of old and new rules over time, and how to adequately manage non-binary antibiotic stewardship classification problems), this example testifies to the potential usefulness of ML for supporting antibiotic stewardship teams that need to simultaneously and continuously evaluate and control prescriptions in several large wards in large hospitals, with the final aims of both optimizing patients’ cure and prevent the emergence and dissemination of difficult-to-treat resistant organisms such as MDR-GNB.

## 7. Conclusions

In this review, we selected and discussed three examples of the interaction between researchers/clinicians dealing with MDR-GNB and ML algorithms. Of course, there are other valuable examples of application of ML to antimicrobial resistance, but it should be noted that our aim was mainly that of introducing the potential usefulness of wisely coupling current research approaches with ML techniques to improve our ability to deal with routine MDR-GNB risk prediction at patients’ bedside.

It is also important to note that we limited our review to ML algorithms trained on clinical (patients’ characteristics and recorded clinical features) and laboratory data (phenotypical identification and susceptibility test) usually available in routine clinical practice in most hospitals. In this regard, the possible dissemination and appropriate use of innovative tests able to provide information on either the precise mechanisms of resistance or other phenotypic/genotypic features of MDR-GNB may further and considerably improve the ability of ML algorithms to help clinicians predicting MDR-GNB risks in routine care [32,33]. Very importantly, this should occur against a background of wide adherence to the FAIR principles (findable, accessible, interoperable, and reusable) connected to the availability of standardized systems [34,35,36,37,38].

In conclusion, in the next few years we expect to witness an increasingly large number of research studies perfecting the application of ML techniques in the field of MDR-GNB infections. Very importantly, this cannot be separated from the availability of a continuously refined and updated ethical framework allowing an appropriate use and sharing of the large datasets of medical data needed to build efficient ML-based support systems.

## Figures and Tables

**Table 1:** Simplified presentation of the possible use of different machine learning algorithms (also based on already existing and well-established statistical models) *.

UNSUPERVISED	SUPERVISED
Clustering and dimensionality reduction	Regression tasks
▪ **SVD (singular value decomposition)**	▪ Linear
▪ **PCA (principal component analysis)**	▪ Polynomial
▪ **K-means**	▪ Decision trees
▪ **Fuzzy k-means**	▪ Random forest
▪ **Hierarchical clustering**	
▪ **Mixture of Gaussians**	
▪ **SOM (self-organizing maps)**	
Association analysis	Classification tasks
▪ **A priori**	▪ Decision trees/random forest
▪ **FP (frequent pattern)-growth**	▪ K-nearest neighbor
Hidden Markov model	▪ Neural networks
	▪ SVM (support vector machines)
	▪ Naive Bayes
	▪ LDA (linear discriminant analysis)

* This is only a fraction of available machine learning algorithms and distinctions in categories are not absolute. For example, some techniques presented for classification problems in the table may also be used for regression problems, and some algorithms/techniques may be used for both supervised and unsupervised objectives.
